# Felix Stern (1884− 1942)

**DOI:** 10.1007/s00415-026-14141-0

**Published:** 2026-09-19

**Authors:** Jürgen Schlegel, Gabriele Stumm, Kaspar Matiasek, Dennis Tappe

**Affiliations:** 1 Institute of Neuropathology, University of Ulm, Ulm, Germany; 2 Department of Neuropathology, Institute of Veterinary Pathology, Ludwig-Maximilians-University, Munich, Germany; 3 Institute of Pathology and Molecular Diagnostics, University of Augsburg, Augsburg, Germany; 4 Bernhard Nocht Institute for Tropical Medicine, Hamburg, Germany

Alongside Constantin von Economo (1876–1931), nearly forgotten German neurologist Felix Stern (1884− 1942) is rightly considered one of the foremost experts on encephalitis lethargica. Nevertheless, little is known about his life and death. Basic information about his biography comes from Aniko Szabo's work on dismissed and persecuted Göttingen university teachers [1] and from research by science journalist Christian Honey published in the German edition of *Scientific American* and contributed to the memorial page for Felix Stern [2].

Felix Stern (Fig. 1) was born on April 5, 1884, in Groß Glogau (now Glogów, Poland) in Lower Silesia and grew up in Berlin, where he attended school and studied medicine. He received his doctorate in Freiburg and then began his clinical and scientific training as an assistant physician in neurology in Kiel. In 1913, he qualified as a professor with a thesis on psychiatric disorders associated with brain tumors. In 1920, he took up an associate professorship with the position of senior physician at the psychiatric clinic in Göttingen. Over time, he saw no further opportunities for advancement in Göttingen, and the salary did not seem particularly attractive either.

**Fig. 1:**
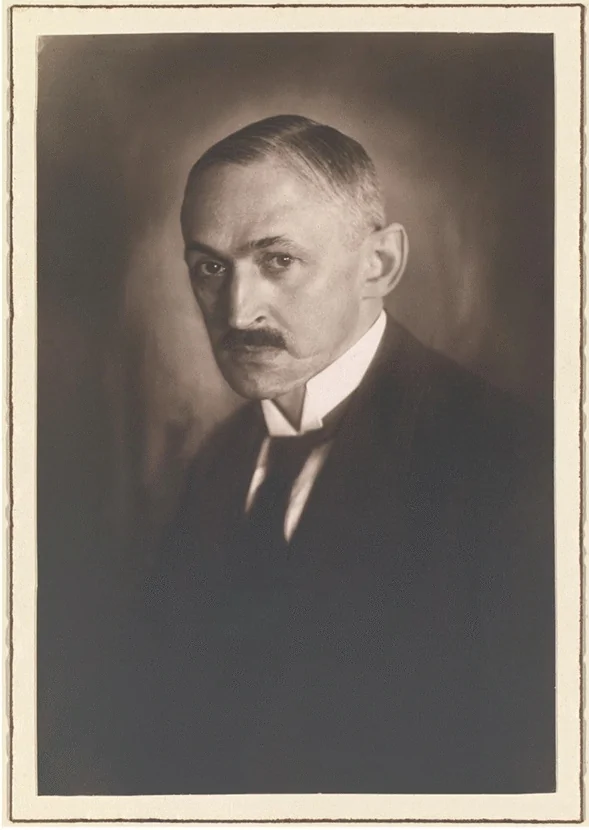
Felix Stern (1884–1942). Courtesy of the Göttingen State and University Library (signature: SUB Göttingen, Voit Collection: Felix Stern, No. 1)

Therefore, in 1928, at the age of 44, he decided to move to Kassel to take up a civil service position as a medical officer in charge of neurology at the medical examination center. He retained his associate professorship in Göttingen and, according to his own statements, fulfilled his teaching obligations at least during the winter semesters.

Stern published a long list of scientific papers, but central were his contributions to the nosological classification of encephalitis lethargica, making him one of the leading experts in the field. Encephalitis lethargica remains one of the most mysterious diseases in medical history [3]. It spread 100 years ago, between 1916 and the late 1920s, as an epidemic encephalitis over the world with hundreds of thousands of deaths. It was the last pandemic for which we ultimately have no idea about the causative infectious agent or the exact pathogenesis of the disease and its sequelae [4].

Stern published his first papers on encephalitis lethargica while still in Kiel. In his first monograph, he described the pathology in detail [5]. Two years later, already in Göttingen, he summarized the clinical aspects in his monograph on “*Die epidemische Encephalitis*” (The epidemic encephalitis) [6]. This monograph appeared in a second, completely revised edition in 1928 [7], now based on his clinical experience with over 800 patients and autopsies of 22 cases in the acute and chronic phase of the disease; the largest collection of encephalitis lethargica cases so far. Regarding the pathogenetic classification of the disease, he was convinced of the infectious origin of a previously unknown virus. He recognized the nosological classification of encephalitis lethargica as a pathological entity, confirmed by the increasing number of his own anatomical examinations [7]. Finally, he wrote the chapter on “Epidemic Encephalitis (Economo’s Disease)” in Bumke and Foerster's *Handbook of Neurology*, which was published in 1936, three years after he was banned from practicing medicine [8]. With this monograph, Stern presented a comprehensive textbook of encephalitis lethargica. It also represents a milestone in the pathogenetic classification of inflammatory diseases of the CNS in general. Stern stated: “The study of encephalitic diseases has undergone a complete revolution thanks to Economo’s discovery of encephalitis lethargica and the study of epidemic encephalitis in general. (…) It was epidemic encephalitis that showed us, as we can say today, that there is a nosological entity in the field of brain inflammation.” [8]

This was a new concept, since the majority of approaches to classify inflammatory diseases of the CNS followed, in principle, Oppenheim and Cassirer’s monograph [9] and were based on a traditional holistic approach. They stated that encephalitis is more likely a spectrum disorder, and therefore, attempts to classify it should be only based on its clinical symptoms. In contrast, Stern further developed the modern pathogenetic approach [10]. With this neuropathological classification, he laid the foundation for understanding infectious and inflammatory diseases of the CNS as nosological entities.

Despite Stern’s recognition in Germany as a leading authority on encephalitis lethargica, his international reputation lagged significantly behind. On the one hand, the encephalitis lethargica pandemic had already declined in 1936 and was no longer a challenging medical problem [4].

On the other hand, Stern's work was not translated into English and therefore did not receive international recognition. This ultimately became a personal problem for him, as despite his significant achievements, he was unable to find a scientific position outside Germany. Felix Stern was a Prussian Protestant, but of Jewish ancestry. Immediately after the Nazis seized power in 1933, he lost his position as a medical officer in Kassel due to „the Law for the Restoration of the Professional Civil Service “, and his teaching license in Göttingen was revoked. He therefore moved to Berlin, where he opened a private practice in 1935. He later continued to practice in his private apartment, where other family members also found refuge.

As early as 1933, while still in Kassel, he submitted his first application to the Academic Assistance Council (AAC), a British-based organization that sought to find positions outside Germany for persecuted scientists. Stern, now 49 years old, sought a clinical or scientific position in the UK, the USA, or elsewhere, and despite the dignity of his applications, they also reveal a certain desperation. Unfortunately, no position could be found for him. The AAC was followed by the Society for the Protection of Science and Learning (SPSL), but his further inquiries there remained unsuccessful. The last documented inquiry to the SPSL dates from 1939. After that, the society received no further communication, so in 1943, the secretary inquired about his whereabouts from colleagues who had emigrated. However, no one was able to provide any information about him. It was not until 1948 that the American Joint Distribution Committee informed the SPSL that Felix Stern was not among the survivors of the Jewish community of Berlin. Felix Stern had committed suicide on August 30, 1942, to escape imminent deportation by the Nazis after members of his family had already been deported.

## Data Availability

Not applicable
